# Association between community garden participation and fruit and vegetable consumption in rural Missouri

**DOI:** 10.1186/1479-5868-10-128

**Published:** 2013-11-19

**Authors:** Ellen K Barnidge, Pamela R Hipp, Amy Estlund, Kathleen Duggan, Kathryn J Barnhart, Ross C Brownson

**Affiliations:** 1 Prevention Research Center in St. Louis, Saint Louis University College for Public Health & Social Justice, 3545 Lafayette Ave., St. Louis, MO 63104, USA; 2 Prevention Research Center in St. Louis, Brown School, Washington University in St. Louis, Campus Box 1006, 621 North Skinker Blvd., St. Louis, MO 63130, USA; 3 Division of Public Health Sciences and Alvin J. Siteman Cancer Center, Washington University School of Medicine, Washington University in St. Louis, St. Louis, MO, USA

**Keywords:** Community gardens, Nutrition, Rural population

## Abstract

**Background:**

Fruit and vegetable consumption reduces chronic disease risk, yet the majority of Americans consume fewer than recommended. Inadequate access to fruits and vegetables is increasingly recognized as a significant contributor to low consumption of healthy foods. Emerging evidence shows the effectiveness of community gardens in increasing access to, and consumption of, fruits and vegetables.

**Methods:**

Two complementary studies explored the association of community garden participation and fruit and vegetable consumption in rural communities in Missouri. The first was with a convenience sample of participants in a rural community garden intervention who completed self-administered surveys. The second was a population-based survey conducted with a random sample of 1,000 residents in the intervention catchment area.

**Results:**

Participation in a community garden was associated with higher fruit and vegetable consumption. The first study found that individuals who worked in a community garden at least once a week were more likely to report eating fruits and vegetables because of their community garden work (X^2^ (125) = 7.78, p = .0088). Population-based survey results show that 5% of rural residents reported participating in a community garden. Those who reported community garden participation were more likely to report eating fruits 2 or more times per day and vegetables 3 or more times per day than those who did not report community garden participation, even after adjusting for covariates (Odds Ratio [OR] = 2.76, 95% Confidence Interval [CI] = 1.35 to 5.65).

**Conclusion:**

These complementary studies provide evidence that community gardens are a promising strategy for promoting fruit and vegetable consumption in rural communities.

## Background

Fruit and vegetable consumption reduces chronic disease risk [1,2], yet the majority of Americans do not meet current consumption recommendations [3]. Although individual and interpersonal determinants are important, there is an increased emphasis on environmental factors that influence fruit and vegetable consumption. One specific environmental strategy - community gardens - is gaining attention for the potential to increase the availability of, and access to, fruits and vegetables [4,5].

Community gardens are associated with increased community connectedness and civic engagement, but few studies examine the effect of community garden participation on fruit and vegetable consumption [6-9]. McCormack and colleagues identified only 4 cross sectional studies examining this relationship in the United States [7,8,10-12]. Each study found a significant association between community garden participation and fruit and vegetable consumption; however, methodological issues such as use of non-validated measures, convenience samples, or lack of pre-intervention measures limit the findings. A more recent study in Denver, Colorado addressed some of these methodological issues: using an in-person population-based survey, the researchers found that those who participated in an urban community garden consumed more fruits and vegetables per day than those who did not participate in a community garden [6].

The existing data supporting this association is promising yet limited to urban settings. To the authors’ knowledge, there are no published studies examining this relationship in rural settings in the United States. Rural settings are of particular interest for several reasons. Rural residents are less likely to meet recommendations for fruit and vegetable consumption than suburban and many urban residents [13]. Rural areas have higher poverty rates than urban and suburban areas [14]. A recent study found that while the majority of rural residents live within 10 miles of a grocery store, low-income rural residents are more likely to live 10 or more miles from a grocery store than middle and high income rural residents [15]. High poverty rates coupled with limited access to a grocery store may explain why rural residents are less likely to meet recommendations for fruit and vegetable consumption. Identifying intervention strategies that increase access to and availability of fruits and vegetables for low income rural residents is needed. The purpose of this study is to examine the relationship between community garden participation and fruit and vegetable consumption in rural Missouri.

### Overview of the intervention

Healthier Missouri Communities (Healthier MO) is a community-based research project conducted by the Prevention Research Center in St. Louis (PRC-StL) and community partners from 12 counties in rural southeast Missouri. The impetus to work in partnership with southeast Missouri communities is the high poverty rate (approximately 20%, nearly double the Missouri rate of 11.8%) and a significantly higher burden of chronic disease than the rest of the state [16]. Compared to the US average, fewer Missouri residents eat fruit 2 or more times a day (27.3% Missouri v. 32.5% US) or vegetables 3 or more times a day (23.0% Missouri v. 26.3% US) [17]. Data suggest that southeast Missouri residents are even less likely to meet recommendations for fruit and vegetable consumption than the state as a whole [18].

Healthier MO seeks to implement environmental and policy interventions to promote healthy eating in this geographic region. In 2010, community partners participated in an evidence-based decision-making training in which they identified community gardens as a feasible and important option to promote fruit and vegetable consumption in their communities. This manuscript reports the findings from two surveys conducted as part of Healthier MO. The community garden intercept survey assessed the effect of frequency of community garden participation in Healthier MO gardens on fruit and vegetable consumption. The population-based survey assessed general community garden participation in the intervention catchment area and the effect of community garden participation on meeting recommendations for fruit and vegetable consumption.

## Methods

This research was approved by the Saint Louis University Institutional Review Board.

### Community garden intercept survey

This study included 12 community gardens, representing seven counties within the 12-county intervention catchment area. As part of the intervention, communities received funding for garden equipment, technical assistance, and access to a regional community garden resource network. The placement of intervention gardens depended on the interest and commitment of each community. Five of the 12 gardens were newly developed for this intervention period. Seven gardens existed prior to the intervention and expanded during the intervention period. The community garden formats varied across the study sites. Half of the community gardens (n = 6) had a single large plot tended collaboratively by multiple gardeners. The other half (n = 6) included multiple individual plots within a larger designated area each tended by individual gardeners. Gardens ranged in size from 147′ to 132′ for the single plot to 4′x20′ for individual plots. Community gardens with individual plots included between six and 40 plots each; 3 of these gardens utilized raised beds as the individual plots. The number of gardeners per garden ranged from three to sixteen. The garden season lasted from approximately May 1st to September 30th.

A quantitative, self-administered, post survey was conducted with a convenience sample of community gardeners from each of the 12 intervention gardens during October of 2011. Gift cards were provided to respondents in appreciation of their participation. The intercept survey included questions about demographics, frequency of working in the community garden, and the self-perceived impact of working in the community garden on behaviors, attitudes, knowledge and skills [19]. Frequency of working in the community garden was dichotomized into “once a week or more” and “less than once a week.” A five-point Likert scale that ranged from “Strongly agree” to “Strongly disagree” was used as response options for questions on behavior, attitudes, knowledge, and skills. Responses were dichotomized as “Yes” if response was “Agree” or “Strongly Agree,” and “No” if response was “Neutral,” “Disagree,” or “Strongly Disagree.” “Don’t know” and “refused” responses were excluded from analysis.

### Population-based telephone survey

The Survey Research Laboratory at Mississippi State University conducted 1,000 telephone interviews with adult respondents from the following towns in the 12-county intervention catchment area: Charleston, Ellington, West Plains, Mountain View, and Doniphan. The towns were chosen because they each have a community garden within a five mile radius. Household telephone numbers were selected from a random-digit-dial sample of 16,000 landline numbers within a five-mile radius of the latitude and longitude coordinates for each town. The sample included households with unlisted numbers. The total number of completed surveys from each location was stratified according to 2009 population estimates. Interviewers collected data during October and November 2011.

Interviewers asked respondents about demographic characteristics based on questions from the 2009 Behavioral Risk Factors Surveillance Survey (BRFSS) questionnaire [20]. The research team assessed respondents’ perceptions of their social and physical environments across three domains: sense of belonging, social cohesion, and food environment. Respondents were asked to answer questions on social and physical environment using a five-point Likert scale that ranged from “Strongly agree” to “Strongly disagree”. The sense of belonging scale included items such as “my community is a good place for kids to grow up” and “I expect to live in this community for a long time [21].” The social cohesion scale included items such as “people around here are willing to help their neighbors,” and “this is a close knit community [22].” The food environment scale assessed ease of buying fresh fruits and vegetables in their neighborhood, quality of fresh produce, selection of fresh produce, ease of buying low fat products, quality of low fat products, and selection of low fat products [23]. Scores for each scale were summed for component questions within each domain to produce a composite domain score, with higher domain scores reflecting stronger sense of belonging, social cohesion, and food environment.

The research team assessed community garden participation based on questions developed by Litt and colleagues for measures of garden participation in Denver [6]. Community garden was defined as a garden where land is shared by others. Community garden participation was defined as growing fruits and vegetables in a community garden and/or receiving fruits and vegetables from a community garden in the last six months; all others were coded as non-participants of community gardens.

Fruit and vegetable consumption was measured using six items from the 2009 BRFSS [20] that determine the frequency of consumption of specific fruits and vegetables per day, week, month, or year. A composite measure of fruit and vegetable consumption was calculated to determine the typical number of times participants consumed fruits and vegetables per day. The composite measure was dichotomized into those who reported consuming “fruit 2 or more times a day and vegetables 3 or more times a day” (meeting recommendations) and those who do not meet this recommendation.

#### Analysis

The research team performed all analyses using SAS version 9.3 (SAS Institute, Cary, NC). Descriptive statistics were used to examine study population characteristics for each survey. Both surveys were used to examine the effect of community garden participation (independent variable) on fruit and vegetable consumption (dependent variable).

### Community garden intercept survey

Because this survey was conducted on a sample of known community gardeners, community garden participation (independent variable) was based on self-reported frequency of working in the community garden: once a week or more vs. less than once a week. Meanwhile, fruit and vegetable consumption was determined by the response to “Because I work in the community garden, I eat more fruits and vegetables” (dependent variable). Responses to other behavior, attitudes, knowledge, and skills questions were secondary outcomes. Chi-square analyses were conducted to estimate the association between more frequent participation in a community garden (once a week or more) with each primary and secondary outcome.

### Population-based survey

For the population-based survey, community garden participation (independent variable) was based on self-reported community garden participation (grows fruits and vegetables in a community garden, or obtained fruits and vegetables from a community garden in past 6 months) vs. non-participation. Fruit and vegetable consumption (dependent variable) was based on a composite measure estimating consumption of eating fruits 2 or more times a day and eating vegetables 3 or more times per day (reflecting meeting and not meeting the daily fruit and vegetable recommendations). A series of multivariate logistic regression models were used to examine the relationship between community garden participation and fruit and vegetable consumption with and without adjustment for covariates. Model 1 examines the effect of community garden participation alone on fruit and vegetable consumption. Model 2 adds sociodemographic covariates: gender, race/ethnicity, age, and education. Model 3 adds sociodemographic covariates as well as social and physical environment domains: social cohesion, sense of belonging, and food environment.

## Results

### Community garden intercept survey

One hundred and forty-one adult community gardeners completed the survey. Participants in the intercept survey were mostly women (67.4%) and mostly Non-Hispanic White (54.6%) or African American (34.8%) (Table 1). Most were 45 years of age or older (72.3%) and more than half had more than a high school education (53.2%). Sixty-four percent of the survey participants reported working in a community garden once a week or more.

**Table 1:** Sociodemographic characteristics of intercept survey respondents in rural Missouri (N = 141)

**Characteristic**		**n (%)**
Gender	Male	40 (28.4)
	Female	95 (67.4)
	Missing	6 (4.3)
Age	18-24	4 (2.8)
	25-44	27 (19.1)
	45-64	78 (55.3)
	65+	24 (17.0)
	Missing	8 (5.7)
Race/ethnicity	Non-Hispanic African American	49 (34.8)
	Non-Hispanic White	77 (54.6)
	All other categories	8 (5.7)
	Missing	(7) 5.0
Education	More than high school equivalency	75 (53.2)
	High school equivalency or less	56 (39.7)
	Missing	10 (7.1)
Community garden exposure	Once a week or more	90 (63.8)
	Less than once week	42 (29.8)
	Missing	9 (6.4)

There is a significant relationship between frequency of community garden participation and perception of consuming more fruits and vegetables because of their community garden work (X^2^ (125) = 7.78, p = .0088) (Table 2). Community gardeners reporting participation once a week or more were more likely to perceive eating more fruits and vegetables.

**Table 2:** Bivariate associations between frequency of community garden work and changes in behaviors, attitudes, and skills for intercept survey respondents

		***Garden frequency***		
		**Once a week or more**	**<Once a week**	
	**N**	**n (%)**	**n (%)**	**Chi sq.**
*Primary outcome:*				
I eat more vegetables and fruit	125	82 (65.6)	31 (24.8)	7.78*
*Secondary outcome:*		a		
I eat food that is fresher (less packaged food)	124	84 (67.7)	27 (21.8)	15.38
I am more physically active	121	74 (61.2)	20 (16.5)	14.48
I spend less money on food	112	66 (58.9)	20 (17.9)	10.17
I am better able to provide food for my family and myself	120	70 (58.3)	25 (20.8)	7.95
I care more about the environment	125	73 (58.4)	27 (21.6)	5.74
I eat less fast food	120	64 (53.3)	22 (18.3)	5.19
I feel better about where my food comes from	119	81 (68.1)	30 (25.2)	4.52
I feel more involved in this neighborhood	124	80 (64.5)	32 (25.8)	4.45
I am teaching my family/friends to garden	123	66 (53.7)	24 (19.5)	3.94
I spend more time with my family	123	55 (44.7)	19 (15.4)	3.12
I have learned more about gardening	126	78 (61.9)	32 (25.4)	2.82
I have gained new gardening skills	128	74 (57.8)	30 (23.4)	2.58
I am donating/giving extra food to others	126	77 (61.1)	32 (25.4)	2.13
I know more about the environment	124	70 (56.5)	28 (22.6)	1.80
I eat more foods that are traditional for my culture/family background	120	53 (44.2)	20 (16.7)	1.03
I eat new kinds of food	118	58 (49.2)	24 (20.3)	0.54

*25% of the cells for this cross-tabulation have expected counts < 5; chi-square may not be a valid test. The 2-sided Fisher’s exact p-value is 0.0088.

Those with more frequent community garden participation were also more likely to report the following secondary outcomes as a result of their community garden work: eating food that is fresher (less packaged food), spending less money on food, being better able to provide food for family and self, eating less fast food, caring more about the environment, and feeling better about where one’s food comes from (Table 2).

### Population-based survey

Participants in the population-based survey were mostly women (73.4%) and non-Hispanic whites (88.0%) (Table 3). Most were 45 years of age or older (81.2%) and less than half had more than a high school education (44.2%). Forty-two percent of the participants in the population-based survey reported growing fruits and vegetables at home while 5% of participants reported participation in a community garden (grows fruits and vegetables in a community garden, or obtained fruits and vegetables from a community garden in past 6 months).

**Table 3:** Sociodemographic characteristics of population-based survey respondents (N = 1,000)

**Characteristic**		**%**
Gender	Male	26.6
	Female	73.4
	Missing	0.0
Age*	Mean age = 59.7	99.0
	Missing	1.0
Race/ethnicity	Non-Hispanic White	88.0
	All other categories	11.2
	Missing	0.8
Education	High school equivalency or less	55.4
	More than high school equivalency	43.9
	Missing	0.7
Social cohesion score*	Mean score = 9.7	97.6
	Missing	2.4
Sense of belonging score*	Mean score = 10.1	98.3
	Missing	1.7
Food environment score*	Mean score = 11.3	98.5
	Missing	1.5
Community garden exposure	Does not participate	95.4
	Participates	4.6
	Missing	0.0
Meets daily fruit & vegetable consumption recommendations	Does not meet recommendations	72.8
	Meets recommendations	13.9
	Missing	13.3

*Continuous variable.

Community garden participation was significantly and positively associated with meeting daily fruit and vegetable recommendations (consumption of fruit 2 or more times a day and vegetables 3 or more times a day) in all three statistical models (ORs ranged from 2.70 to 2.76). The effect sizes for this association were consistent across all three models, with community garden participation associated with a more than two-fold increase in likelihood for meeting daily fruit and vegetable recommendations in the final fully adjusted model (OR = 2.76, 95% CI: 1.35 to 5.65) (Table 4).

**Table 4:** Logistic regression of the association between community garden exposure and recommended daily servings of fruits and vegetables in a population-based survey

**Characteristic**		**OR**	**95% CI**	**P**
Community garden participation	Participates	2.76	1.35 to 5.65	0.0054
	Does not participate	1.0	--	--
Gender	Male	0.67	0.43 to 1.05	0.0822
	Female	1.0	--	--
Age				
Race/ethnicity	Non-Hispanic White	1.63	0.80 to 3.32	0.1765
	All other categories	1.0	--	--
Education	More than high school equivalency	1.18	0.80 to 1.75	0.4130
	High school equivalency or less	1.0	--	--
Social cohesion		1.01	0.95 to 1.07	0.1523
Sense of belonging		1.00	0.97 to 1.04	0.7976
Food environment		0.96	0.90 to 1.02	0.8899

Further regression analyses were performed on the population-based survey disaggregating those who work in the community garden (2.3%) and those who receive fruits and vegetables from a community garden (3.9%). Results show that working in a community garden was not significantly associated with increased fruit and vegetable consumption but the three models trended in the right direction (ORs ranged from 1.56 to 1.78). Obtaining fruits and vegetables from a community garden was significantly associated with fruit and vegetable consumption in all three models and odds ratios were similar to the results for examination of any participation in a community garden.

## Discussion

Together, the findings from our community garden intercept survey and the population-based survey demonstrate an association between community garden participation and fruit and vegetable consumption in rural settings. The intercept survey was a post evaluation of a community garden intervention with known community gardeners. Despite the limitations of the post evaluation only design, our findings suggest that frequent participation in a community garden has greater impact on community gardeners’ perception that they consume more fruits and vegetables. The population-based survey was a random sample that allowed us to estimate general community garden participation in rural settings and compare those who participate in community gardens to those who do not. The association between community garden participation and fruit and vegetable consumption in the population-based survey was robust after adjustment for covariates that reflected both sociodemographic factors and participants’ perception of the social and physical environments.

The findings from these studies contribute to the literature in several ways. To the authors’ knowledge this is the first study to examine the relationship between community garden participation and fruit and vegetable consumption in rural settings. There is one known published study exploring the feasibility and perceived benefit of developing a community garden in a rural African American community in Virginia [24]. Its findings indicated that participants believe a community garden would increase fruit and vegetable consumption among residents and youth and perceived that working in a community garden would increase one’s willingness to try new fruits and vegetables [24]. Although this information is necessary when considering community interest in an intervention strategy, it does not address intervention effectiveness. Data from the studies presented in this article begin to build the evidence.

The studies presented here examined both frequency and types of community garden participation which differs from other studies. The intercept survey examined frequency of community garden participation while most studies compare participation versus non-participation like the population-based survey [6-8]. Unlike other studies that define community garden participation as growing fruits or vegetables in a community garden [6,8], the population-based survey included those receiving fruits and vegetables from a community garden. Together, these studies allow us to consider whether working in a community garden and the frequency of doing so affect consumption. The findings are mixed. The intercept survey found that those who participate more often in a community garden are more likely to report a connection between eating more fruits and vegetables and community garden participation. Further regression analyses were performed on the population-based survey disaggregating those who grow fruits and vegetables in a community garden and those who simply obtain fruits and vegetables from a community garden. The results show that working in a community garden alone was not significantly associated with increased fruit and vegetable consumption; however, the models trended in the right direction and may not have been statistically significant due to small numbers (only 2.3% reported working in a community garden). Obtaining fruits and vegetables from a community garden was significantly associated with fruit and vegetable consumption after controlling for covariates. This suggests that community garden impact on increased fruit and vegetable consumption may be a function of access to produce as much as it is about having a communal place to grow produce.

### Limitations

There are several limitations to note. The intercept survey was designed to be brief as it “intercepted” community gardeners in action. As a result, a single item was used to assess fruit and vegetable consumption. The wording of the question could be leading which introduces bias. Participants may have been more likely to report a perception of eating more fruits and vegetables due to the wording of the question. The intercept survey was conducted with a convenience sample which may introduce selection bias.

The population-based survey collected data using a random sample of landline telephone numbers. Due to increased reliance on cell phones the population sampled in the population-based survey may not reflect the general population of the target area. The population tended to be older (81.2% of the sample is older than 45 years compared to 43.0% of the catchment area population [25]) and may be more or less likely to participate in community gardens than the general population. Descriptive statistics indicated that 13% of data for the dependent variable were missing due to non-response. Examination of missing value patterns showed that our data were not missing completely at random, indicating the potential for bias if we only included those with non-missing values in our analysis (complete case analysis). Without a complete dataset available for comparison, it is not possible to test for other patterns of missing values (missing at random, or MAR, and missing not at random, or MNAR). Although we were not able to differentiate between MAR and MNAR, we explored a multiple imputation approach that assumes MAR [26]. We specified an imputation model that included all the variables used in analysis and also auxiliary variables [27,28] (specifically, food security status, marital status, county of residence, meets/does not meet physical activity recommendations, and self-rated health status) in order to generate 10 imputed datasets with missing values replaced with reasonable estimates. Analysis was conducted on each imputed dataset and PROC MIANALYZE was used to generate combined odds ratios and 95% confidence intervals that incorporate the uncertainty arising from imputation. We performed sensitivity analysis to examine results both without imputation (complete case analysis) and after multiple imputation under the assumption of MAR. Results were identical in direction and similar in magnitude. We therefore presented only results from complete case analysis here because the similarity of results observed in sensitivity analysis suggest the missing values were not a source of bias.

We measured fruit and vegetable consumption differently in each survey and only captured cross sectional data from a rural area of Missouri. Because we used two separate measures of fruit and vegetable consumption, comparisons of the two surveys is limited. Cross sectional data limits the ability to determine causality. It is plausible that those who participate in community gardens or participate more frequently are already eating more fruits and vegetables than those who do not participate in community gardens. We collected data for both surveys from a 12 county area in rural southeastern Missouri. The results presented here may not reflect associations in other rural communities.

More information on accessibility and distribution of community garden produce is needed. Healthier MO community garden distribution practices varied. Some gardens allowed relatively open access to produce regardless of participation in growing while others restricted access to only those who grew the produce. Others have suggested that when community food infrastructure is designed to accommodate the needs of the poor, such as open access to community gardens, dietary behavior change may result [4]. One way to increase the reach of community gardens is to distribute the produce grown beyond the gardeners. A second consideration of accessibility of produce grown in community gardens is the location of the gardens to the population. As noted earlier low income rural residents are more likely to live further away from grocery stores. This may be an important limitation of the effectiveness of community gardens in rural areas as well. The research team for this study did not collect data on distance to a community garden. Community garden location is an important consideration given transportation needs and barriers for some rural residents. It is important to note that the focus on community gardens was chosen by rural residents participating in Healthier MO who prioritized community gardens as an environmental strategy that was both important and feasible for their communities.

## Conclusions

The findings summarized here suggest that community gardens may be an effective environmental strategy to promote fruit and vegetable consumption in rural communities. Public health practitioners should consider participants’ level of participation in a community garden as well as informal policies about who has access to the produce grown if community gardens are developed as a community resource. One of the advantages of community gardens is that the food grown can be distributed to a wider population than those immediately involved. It can therefore reach more people in the community and has the potential to create a ripple effect. As the evidence for community gardens as an environmental strategy to promote fruit and vegetable consumption builds, the next step is to conduct additional rigorously designed studies, and if associations are replicated, develop a systematic approach for scaling up this intervention [29].

## Competing interests

The authors declare that they have no competing interests.

## Authors’ contributions

EB, AE, KD, RB were involved in the concept and study design. EB, AM, KD, and KB collected the data. EB and PRH conducted data analysis and drafted the manuscript. AE, KD, KB, and RB critically revised the manuscript. All authors have read and approved the final version being submitted to the journal.
